# Incidentally identified genetic variants in arrhythmogenic right ventricular cardiomyopathy‐associated genes among children undergoing exome sequencing reflect healthy population variation

**DOI:** 10.1002/mgg3.593

**Published:** 2019-04-15

**Authors:** Andrew T. Headrick, Jill A. Rosenfeld, Yaping Yang, Hari Tunuguntla, Hugh D. Allen, Daniel J. Penny, Jeffrey J. Kim, Andrew P. Landstrom

**Affiliations:** ^1^ Department of Pediatrics, Section of Pediatric Cardiology Baylor College of Medicine Houston Texas; ^2^ Department of Molecular and Human Genetics and Baylor Genetics Laboratories Baylor College of Medicine Houston Texas; ^3^ Department of Pediatrics, Division of Pediatric Cardiology Duke University School of Medicine Durham North Carolina; ^4^ Present address: Department of Pediatrics, Division of Pediatric Cardiology Duke University School of Medicine Duke University Medical Center Durham North Carolina

**Keywords:** arrhythmogenic right ventricular cardiomyopathy, genetic testing, genetics, incidental finding, secondary finding, variant of undetermined significance, whole exome sequencing

## Abstract

**Background:**

With expanding use of clinical whole exome sequencing (WES), genetic variants of uncertain significance are increasingly identified. As pathologic mutations in genes associated with arrhythmogenic right ventricular cardiomyopathy (ARVC) carry a risk of sudden death, determining the diagnostic relevance of incidentally identified variants associated with these genes is critical.

**Methods:**

WES variants from a large, predominantly pediatric cohort (*N* = 7,066 probands) were obtained for nine ARVC‐associated genes (Baylor Miraca). For comparison, a control cohort was derived from the gnomAD database and an ARVC case cohort (*N* = 1,379 probands) was established from ARVC cases in the literature. Topologic mapping was performed and signal‐to‐noise analysis was conducted normalizing WES, or case variants, against control variant frequencies. Retrospective chart review was performed of WES cases evaluated clinically (Texas Children's Hospital).

**Results:**

Incidentally identified variants occurred in 14% of WES referrals and localized to genes which were rare among ARVC cases yet similar to controls. Amino acid‐level signal‐to‐noise analysis of cases demonstrated “pathologic hotspots” localizing to critical domains of PKP2 and DSG2 while WES variants did not. PKP2 ARM7 and ARM8 domains and DSG2 N‐terminal cadherin‐repeat domains demonstrated high pathogenicity while normalized WES variant frequency was low. Review of clinical data available on WES referrals demonstrated none with evidence of ARVC among variant‐positive individuals.

**Conclusions:**

Incidentally identified variants are common among pediatric WES testing with gene frequencies similar to “background” variants. Incidentally identified variants are unlikely to be pathologic.

## 1 INTRODUCTION

Arrhythmogenic right ventricular cardiomyopathy (ARVC) consists of progressive fibrofatty replacement of cardiac myocytes, with preference for the right ventricle, followed by scarring and development of cardiac dysfunction and a substrate for potentially fatal arrhythmias. The most common presenting symptom is ventricular tachycardia, and up to half of cases may present with sudden cardiac death (SCD) (Landstrom, Tester, & Ackerman, 2011; Quarta et al., 2011). ARVC accounts for ~13% of all SCD, despite occurring in only one out of every 5,000 persons, a rate one‐tenth that of the most prevalent inherited SCD‐potentiating cardiomyopathy, hypertrophic cardiomyopathy (HCM) (Finocchiaro et al., 2016).

ARVC is a diagnostically challenging disease due to its heterogeneous clinical presentation (Ackerman et al., 2011). Believed to be heritable, 30%–50% of patients with clinical ARVC host at least one presumably causative variant, and genetic testing is critical for identifying at‐risk family members (Marcus, Edson, & Towbin, 2013). These variants localize to genes encoding components of the cardiac desmosome which is responsible for physically joining the myofilaments of contiguous cardiac myocytes and facilitating cell‐to‐cell transmission of contractile force. Hundreds of putative variants have been identified across all five genes encoding proteins of the cardiac desmosome, and there is a growing list of genes encoding proteins with various indirect roles in desmosomal maintenance. The most common genes associated with ARVC are *PKP2 *(OMIM 602861), *DSP *(OMIM 125647), *DSG2 *(OMIM 125671), *DSC2* (OMIM 125645), *JUP *(OMIM 173325), and *TMEM43 *(OMIM 612048). Less common genetic causes include *TGFB3 *(OMIM 190230), *PERP *(OMIM 609301), *PKP4 *(OMIM 604276), as included in this study, as well as *CTNNA3 *(607667), *DES *(OMIM 125660), *LMNA *(OMIM 150330), *PLN *(OMIM 172405), *CDH2 *(OMIM 114020), and *TTN *(OMIM 188840), among others (McNally, MacLeod, & Delleface‐Castillo, 2017).

While guidelines for genetic testing for ARVC recommend the use of gene panels, the use of clinical whole exome sequencing (WES) has dramatically increased (Ackerman et al., 2011). Clinical WES testing has proven helpful in identifying genetic etiologies of disease when clinical presentations are heterogenous or atypical, this has led to a dramatic increase in incidentally identified variants of uncertain significance (VUS) (Meng et al., 2017; Yang et al., 2013). This is most significant in children where the lifetime risk of cardiomyopathy development, in the setting of an incidental genetic finding, remains unknown. Several studies have documented a “background” rate of rare variants in ARVC‐associated genes among ostensibly healthy individuals or population‐based genetics studies. However, these studies do not address the incidental findings of a child who underwent WES testing for noncardiac indications yet identifying a variant in an ARVC‐associated gene. To our knowledge, there has been no study which directly addresses the diagnostic value of incidentally identified variants in ARVC‐associated genes.

In this study, we hypothesized that incidentally discovered variants in ARVC‐related genes found in children with low pre‐test suspicion of ARVC represent healthy genetic background noise. We compared incidentally identified variants in a large WES referral cohort against a cohort of pathologic cases and a cohort of population‐based controls. Utilizing variant mapping and signal‐to‐noise (S:N) analyses, we demonstrate marked overlap in WES and control variants. Finally, we retrospectively reviewed the clinical data and imaging of WES cohort subjects evaluated at Texas Children's Hospital (TCH) and found no clinical evidence of ARVC.

## 2 METHODS

### 2.1 Ethical compliance

This research study was approved by the Baylor College of Medicine Institutional Review Board.

### 2.2 Nomenclature

Study nomenclature is detailed in Supplemental Methods.

### 2.3 Study cohorts

Study cohorts included a WES variant cohort, an ARVC case cohort, and a control variant cohort. To evaluate for clinical evidence of ARVC among WES variant‐positive individuals, the TCH Clinic Cohort was created. These are detailed in Supplemental Methods.

### 2.4 Signal‐to‐noise calculation and topologic mapping

Gene‐ and amino acid‐level (S:N) analyses were conducted as previously described (Landstrom et al., 2018) and are fully detailed in the Supplemental Methods.

### 2.5 Statistics

Fully detailed in the Supplemental Methods.

## 3 RESULTS

### 3.1 Prevalence of rare variants in ARVC‐associated genes among WES referrals

**Figure 1:**
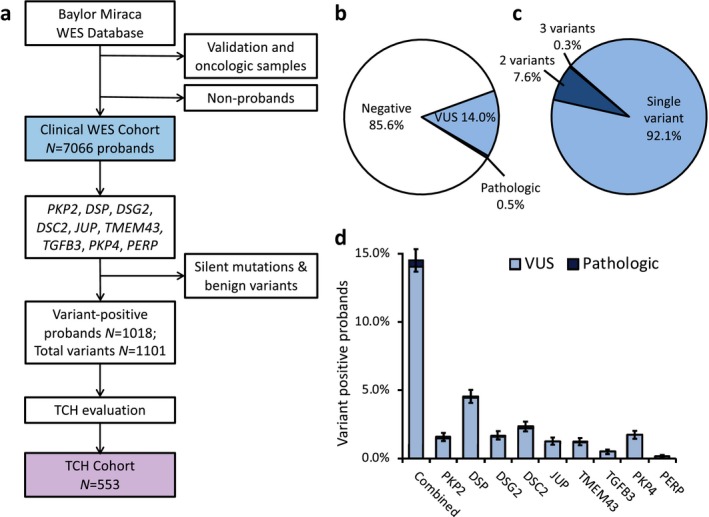
(a) Schematic of derivation of WES cohort. The clinical WES cohort comprised of variants eligible for clinical reporting excluding test validation studies and oncological samples. Silent variants and variants interpreted as benign were excluded. (b) Pie chart demonstrating breakdown of variant negative subjects (white fill), and individuals with a variant interpreted as pathologic (dark blue fill) or VUS (light blue fill), at time of testing. (c) Pie chart of WES cohort individuals with at least one positive variant. (d) Stacked column graph of WES cohort variant frequency by gene and by VUS (light blue fill) versus pathologic (dark blue fill) for respective genes. TCH, Texas Children's Hospital. VUS, variant of uncertain significance. WES, whole exome sequencing

To determine the spectrum and frequency of incidentally identified variants in ARVC‐associated genes, the WES cohort was compiled (Figure 1a). This cohort consisted of 7,066 probands, with 1,018 (14.4% [13.6–15.2]) individuals found to have at least one rare variant in any of nine ARVC‐associated genes. A total of 988 (14.0 % [13.2‐14.8], Figure 1b) individuals in the cohort hosted a VUS, and VUSs represented a majority of the total incidentally identified variants (988, 97.1% [96.0‐98.1]). The majority of variant‐positive probands, 938 (92.1% [90.5–93.8]), hosted only one variant, while 77 (7.6% [5.9–9.2]) probands hosted two variants, and three (0.3% [0.0–0.6]) probands hosted three (Figure 1c). The predominance of VUS variants persisted across all genes with no gene showing a significant contribution from variants classified as “likely pathologic” at time of WES genetic testing (Figure 1d). The demographics of this WES cohort, and variant positive individuals within the cohort, are detailed in Table S1. A small number of variants were common among multiple unrelated cases; however, no variant occurred at a frequency of more than 0.6% [0.4–0.8] of the total cohort (Supplemental Results, Table S2). Taken together, these findings show that variants in ARVC‐associated genes are prevalent among WES probands, found in ~14% of all WES referrals, and are most often VUSs.

### 3.2 WES cohort variant frequencies and variant class in comparison to control and ARVC case cohorts

To compare WES‐identified variants with control and ARVC‐afflicted individuals, we next evaluated a control and ARVC case cohort. Among the 138,632 individuals genotyped in the gnomAD, 5,098 unique variants with a MAF < 0.0001 were identified which contributed to a total rare variant MAF of 6.1% [6.0–6.2]. In comparison, the ARVC case cohort consisted of a total of 14 studies from the literature which demonstrated 1,379 ARVC cases with 603 (43.7% [41.1–46.3]) genotype‐positive individuals (Supplemental Results). Some ARVC cohort probands harbored more than one ARVC‐associated mutation, amounting to a cohort variant frequency of occurrence of 46.0% [43.3–48.6]). When normalized against the overall frequency of rare variants among the control cohort, the combined S:N for the ARVC case cohort mutations was ~7.5 compared to ~2.3 in the WES cohort, resulting in a yield that was threefold higher than WES variants.

**Figure 2:**
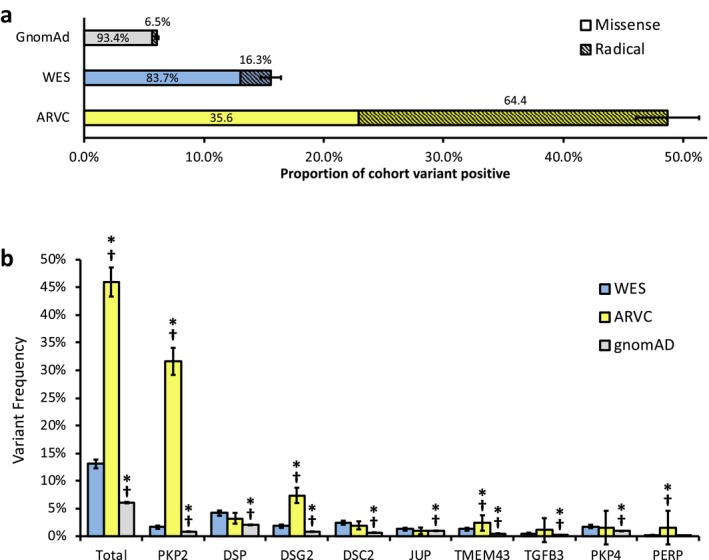
(a) Stacked bar graph demonstrating variant frequency by cohort with gnomAD (light grey fill), WES (light blue fill), and ARVC cases (yellow fill) and respective contributions to such by missense (solid fill) and radical (diagonal stripe fill) variants. Error bars denote 95% confidence interval. (b) Column graph displaying gene dependent variant frequency by cohort. Total column excludes contributions of *PKP4* and *PERP* to each cohort. *p < 0.05, compared to respective gene from WES cohort. ^†^ *p* < 0.05, compared to respective gene from ClinVar‐verified WES cohort (Supplement). Error bars denote 95% confidence interval. ARVC, arrhythmogenic right ventricular cardiomyopathy. WES, whole exome sequencing

There were a number of additional similarities between the WES cohort and control cohort variants. The vast majority of variants within the WES cohort were missense, accounting for 83.7% [81.5–85.8] of total variants, with a similar proportion (93.4% [93.0–94.0]) of variants being missense in the control cohort. The two cohorts displayed relatively low proportions of radical variants (16.3% [14.1–18.5] and 6.5% [6.0–7.0], respectively). In comparison, there was a markedly higher prevalence of radical mutations among ARVC cases, accounting for 64.4% [60.6–68.1] of all identified mutations. This was ~4‐fold higher than the WES frequency and ~10‐fold higher than the control cohort. These results are summarized in Figure 2a. Overall, these results indicate that the frequency of variants in ARVC‐associated genes is markedly lower in both WES and control cohorts when compared to ARVC cases, and variants in ARVC cases show a predilection to radical mutations that is not shared by the WES variants.

### 3.3 Gene‐specific yield with the WES cohort in comparison to control and cases

Given the apparent disparity in global variant frequency across the cohorts, we next evaluated the respective gene‐specific variant frequencies. Again, we identified many similarities between the WES and control cohort. In the WES cohort, variants most commonly localized to *DSP* (4.2% of WES cohort patients [3.7–4.6]), followed by *DSC2* (2.3% [2.0–2.7]), and *PKP4* (1.7% [1.4–2.0]). Similarly, in the control cohort, variants most commonly localized to *DSP* (2.1% [2.0–2.1]), *JUP* (1.0% [0.9–1.0]), and *PKP4* (0.9% [0.9–1.0]). With the exception of *DSP*, many of these genes are traditionally considered to be less common causes of ARVC. In contrast, among ARVC cases, mutations most frequently localized to *PKP2* (31.6% [29.2–34.1]), *DSG2* (7.4% [6.0–8.8]), *DSP* (3.2% [2.3–4.2]), and *TMEM43* (2.4% [1.6–3.2]). There data are summarized in Figure 2b. When ClinVar‐designated “benign variants” were excluded from the WES cohort yielding the ClinVar‐verified WES cohort, there was a statistically significant, but modest reduction in overall WES cohort frequency and frequency of variants found within *DSP* and *DSC2 *(Supplemental Results, Figure S1a). These results demonstrate that variant frequency in the WES cohort, similar to the control cohort, was driven by contributions from variants in genes that are rarely associated with ARVC cases.

**Figure 3:**
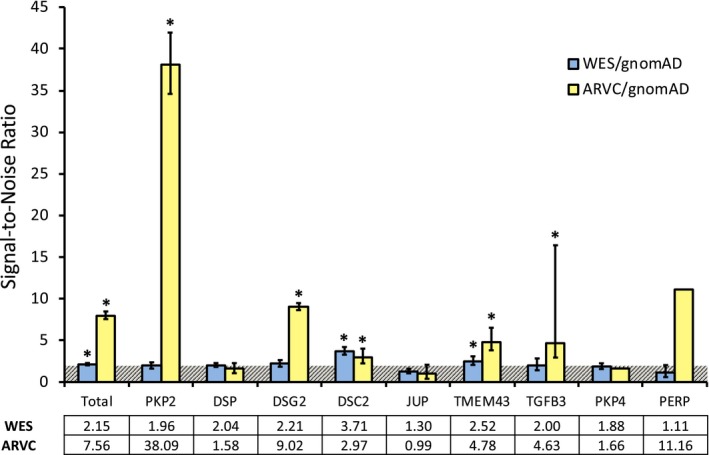
S:N analysis of WES and ARVC cohorts normalized to gnomAD variants. Horizontal bar with diagonal hashing marks 2.0. Total column excludes contributions of *PKP4* and *PERP* to each cohort and variance (error bars) is not included for ARVC/gnomAD due to small sample size. *p < 0.05 above a S:N threshold of 2. Error bars represent 95% confidence interval. ARVC, arrhythmogenic right ventricular cardiomyopathy. WES, whole exome sequencing

Given evidence that disease‐specific S:N calculations can distinguish background genetic noise from true pathologic variant burden, this analysis was applied to each gene locus (Landstrom et al., 2017, 2018). Among ARVC cases, several genes demonstrated S:N ratios significantly greater than 2. The greatest was *PKP2* (38.09 [34.59–41.95]), followed by *DSG2* (9.02 [8.64–9.48]), *TMEM43* (4.78 [3.81–6.53]), and *DSC2* (2.97 [2.25–4.02]), which are traditionally believed to be the most common genes mutated in ARVC. In comparison, among WES cohort variants, *DSC2*, *TMEM43* and *DSG2*‐associated variants demonstrated the greatest S:N ratios, at 3.71 [3.30–4.12], 2.52 [2.06–2.97] and 2.21 [1.85–2.57], respectively. The remaining genes, including *PKP2* and *DSG2*, demonstrated a S:N ratio that was statistically indistinguishable from 2.0 (Figure 3).

### 3.4 Variant amino acid location overlaps between cohorts

**Figure 4:**
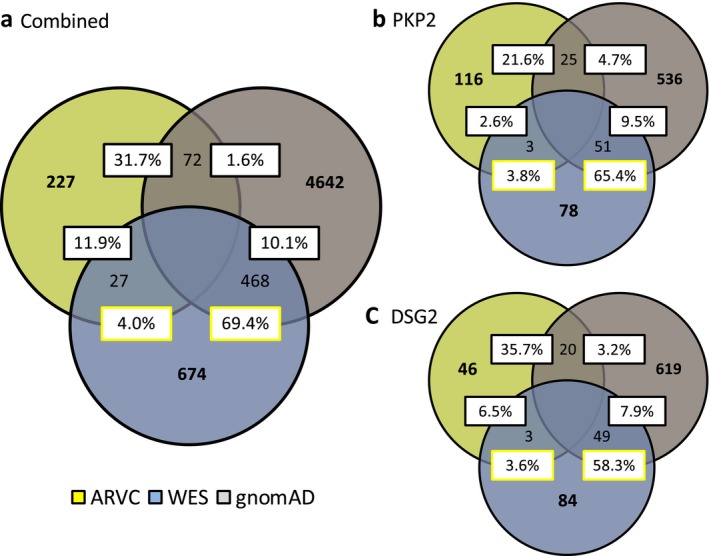
(a) Variant position overlap between study cohorts. Venn diagram of the colocalization of unique variants to residues common to the ARVC case (yellow fill), gnomAD (light grey fill), and WES (light blue fill) cohorts. (b) PKP2 variant position overlaps between study cohorts. (c) DSG2 variant position overlaps between study cohorts. ARVC, arrhythmogenic right ventricular cardiomyopathy. WES, whole exome sequencing

We next evaluated the tendency of WES variants to overlap in affected amino acid position compared to the control and ARVC case mutations. Overall, the WES cohort variants localized to 674 unique amino acid positions along the primary sequence of all analyzed ARVC‐associated proteins, while ARVC case mutations occupied 227 positions, and control variants occupied 4,642. The WES cohort variants shared amino acid positions with only 27 (4.0% [2.5–5.5] of all WES positions) of the ARVC cohort mutation positions. In contrast, the WES cohort showed strikingly similar localization with the control cohort, with 468 (69.4% [66.0–72.9]) amino acid positions held in common between the two cohorts (*p* < 0.00001). The WES‐to‐ARVC relationship was mirrored by the control‐to‐ARVC comparison, in which only 72 unique amino acid positions, or 1.6% [1.2–1.9] of the control cohort, were shared (Figure 4a). This tendency for WES variants to overlap with control amino acid positions, and not ARVC cases, persisted at the individual gene level. Specifically, for PKP2 the WES cohort demonstrated 78 unique affected positions, of which 3 (3.8% [2.4–5.3]) were shared with the 116 of the ARVC case cohort, whereas 51 (65.4% [61.8–69.0]) were shared with the 536 of the control cohort (*p* < 0.00001; Figure 4b). For DSG2*,* only 3 (3.6% [2.2–5.0]) of the 84 unique affected amino acid positions in the WES were shared with the 46 of the case cohort. Conversely, the WES shared 49 (58.3% [54.6–62.1]) positions with the 619 of the control cohort (*p* < 0.00001; Figure 4c). This association was similarly seen in DSP, DSC2, and TMEM43 (Supplemental Results, Figure S2).

### 3.5 Amino acid‐level signal‐to‐noise analysis

**Figure 5:**
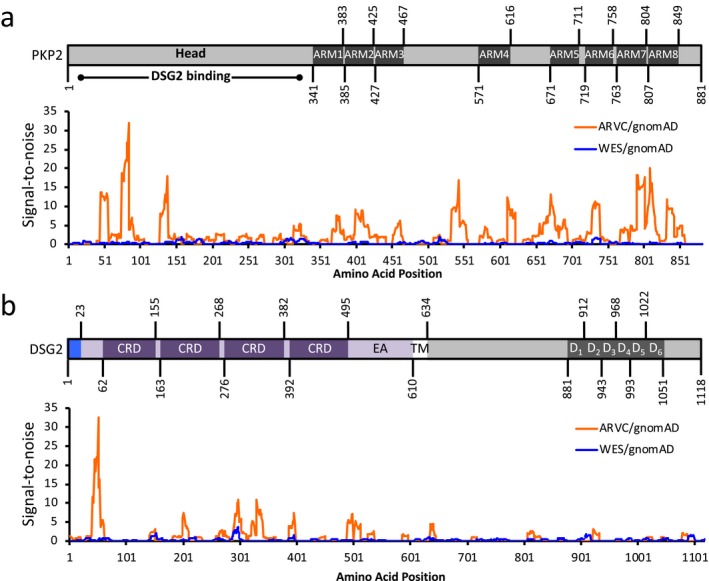
Topological signal‐to‐noise (S:N) mapping between WES (blue line) and ARVC (orange line) cohorts, as normalized against gnomAD for (a) PKP2 (NP_001005242.2), and (b) DSG2 (NP_001934.2). Accompanying protein topologies are shown to scale. Intracellular regions (grey fill), extracellular regions (purple fill), transmembrane regions (white fill), and signal pro‐peptides (light blue fill), are represented. ARM, armadillo/beta‐catenin‐like repeat. ARVC, arrhythmogenic right ventricular cardiomyopathy. CRD, Cadherin repeat domain. D_1–6_, DSG repeat domains 1–6. EA, extracellular anchor. Head, head domain. TM, transmembrane domain. WES, whole exome sequencing

We have previously shown that amino acid‐level S:N can distinguish incidentally identified from pathologic variants based on primary sequence location in genes associated with cardiac channelopathic disease (Landstrom et al., 2017, 2018). We applied this methodology to ARVC‐associated gene products. In PKP2, the ARVC cohort showed significant S:N intensity in several key functional domains of the protein. The two largest peaks occurred in the head domain, a region responsible for DSG2 binding (amino acids 74–92, peak at amino acid 84; 128–145, peak at 138). These two large signals were primarily due to two frequently occurring nonsense mutations with resultant premature truncating proteins PKP2‐R79X and PKP2‐Q133X, both of which have been previously described as recurrent and representing a founder effect in the population (van Tintelen et al., 2006; van der Zwaag et al., 2010). Neither of these variants was present in the WES cohort. The remaining remarkable areas of S:N occurred at several points in the armadillo/beta‐catenin‐like‐repeats regions (ARM), particularly ARM7 and ARM8. Contributing variants are further discussed in the Supplemental Results section. These solenoid protein domains serve many purposes such as facilitating protein‐to‐protein interactions and in the formation of adherens junction complexes (Lancet, Safran, & Rosen, 2018; Povey & McAlpine, 2002). None of these signal areas in the ARM repeat domains correlated with reported founder variants, and were present across multiple studies with different populations. Interestingly, two of the highest peaks occur within the armadillo‐fold region corresponding with an alpha‐helix, but not falling within any reported functional domain (526–553, peak at 543), rather occurring N‐terminal to ARM4. This region lacks any reported protein domain or function at present. There were no such similarly high S:N peaks in the WES cohort variants (Figure 5a).

In DSG2, case mutations were prominently found in the extracellular domains. The largest S:N peak occurred N‐terminal to the first cadherin‐repeat domain (CRD, 30–55, peak at 51) but did not correlate with a specific reported functional domain. Three additional areas of high signal correlated to the remaining three CRDs which are crucial for cell–cell adhesion and thus maintenance of the structural integrity of cardiac tissue (Lowndes et al., 2014). The WES cohort showed one region of signal concentration deviating from S:N of 1.0 and corresponding to the third CRD, beginning at amino acid position 287 and continuing to 299, with a peak S:N ratio of 3.5 at position 296. The five unique missense variants in this region each occurred only once (Supplemental Results, Table S3). Of note, the ARVC cohort demonstrated a corresponding signal in this same region approximately threefold that of the WES (Figure 5b).

### 3.6 Clinical evaluation of referrals with WES‐identified ARVC‐associated gene variants

**Figure 6:**
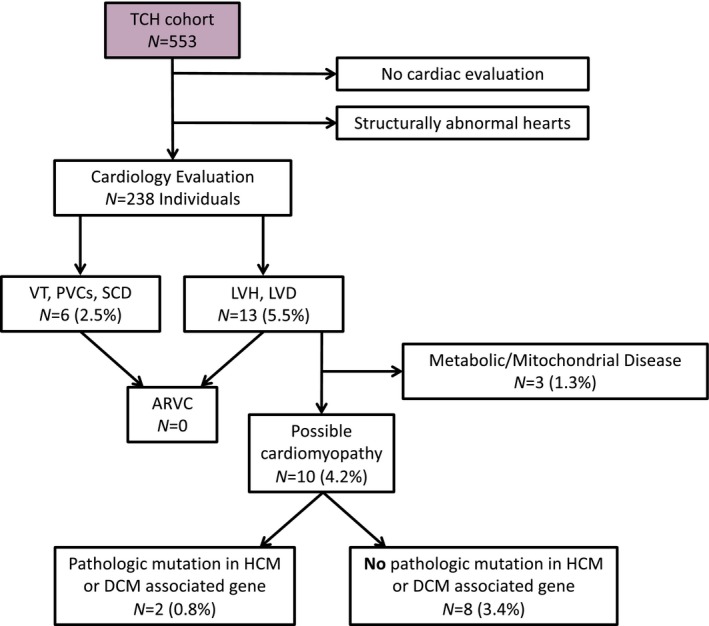
Schematic of TCH clinical subcohort and individuals demonstrating evidence of cardiomyopathy. ARVC, arrhythmogenic right ventricular cardiomyopathy. DCM, dilated cardiomyopathy. HCM, hypertrophic cardiomyopathy. LVD, left ventricular dilation. LVH, left ventricular hypertrophy. PVC, premature ventricular contraction. SCD, sudden cardiac death. TCH, Texas Children's Hospital. VT, ventricular tachycardia

To determine whether any of the incidentally identified WES variants were found in individuals with evidence of ARVC, we retrospectively reviewed clinical records. In total, 553 variant positive subjects in the WES cohort were seen at TCH, 238 of whom were evaluated by cardiology and had an echocardiogram with an average follow‐up of 2.7 [0.0–10.4] years (Figure 6). A small number, 13 (5.5%), of these patients had either left ventricular hypertrophy and/or dilation, six patients (2.5%) had a history of documented ventricular tachycardia or frequent PVCs (premature ventricular contraction), and none had a history of SCD. Of the 13 patients with known cardiomyopathy, three patients had tissue and genetic results consistent with underlying mitochondrial disease, two (0.8%) had known pathologic variants on WES in a hypertrophic or dilated cardiomyopathy‐associated gene. Six (2.5%) had a VUS in a hypertrophic or dilated cardiomyopathy‐associated gene. The details of these patients are summarized in Table S5. No subjects had recorded clinical suspicion or a symptomatic constellation consistent with ARVC.

## 4 DISCUSSION

Genetic testing is an indispensable tool in the evaluation of heritable cardiomyopathies such as ARVC, particularly in identifying clinically‐at‐risk individuals within a family following identification of a likely causative mutation in a proband. This is supported by the finding that ~46% of ARVC cases in our study were positive for a likely causative mutation in an ARVC‐associated gene. ARVC gene panel testing is recommended in patients that meet a borderline or possible diagnosis in accordance with evidence‐based ARVC task force guidelines for clinical diagnosis, most recently updated in 2010 (Marcus et al., 2010; McNally et al., 2017). While the recommended methodology for genetic testing in suspected ARVC endorses the use of gene panels, clinical WES is being increasingly utilized for children with suspected genetic disease. Recent data suggest that up to 25% of heritable diseases, not diagnosed by traditional genetic testing methods, may have underlying genetic explanations revealed by WES (Yang et al., 2014). These advances in genetic testing offer hope for earlier diagnosis prior to symptomatic onset, of particular interest in a ARVC as the early recognition of disease and, when appropriate, placement of an implantable cardiac defibrillator significantly reduces hospitalizations and mortality (Boriani et al., 2007; Komura et al., 2010; Mazzanti et al., 2016). While there is undoubtedly utility to WES testing, the diagnostic value of incidentally identified variants in cardiomyopathy and channelopathy genes has not been clarified in these children.

Complexity in interpreting incidental variants is highlighted by the frequency of control variants. Previous work in ARVC has demonstrated a background rare variant rate of ~16% in Sanger‐sequenced healthy control individuals (Kapplinger et al., 2011). Here, we document a background genetic rare variant frequency of 6.1% within the gnomAD database. Although this is notably less than previously reported, it may serve as a more accurate representation given the large and diverse sample size. Further, our application of a rare variant MAF threshold of <0.0001 was not applied to earlier work given the small cohort size (Kapplinger et al., 2011). Similar genetic background variation has been reported in the primary heritable arrhythmogenic disorders catecholaminergic polymorphic ventricular tachycardia and long QT syndrome, with rates of 6.0% and 12.9%, respectively (Landstrom et al., 2017, 2018). While the background rate of rare variants in ARVC‐associated genes is relatively low, approximately 1 in every 20 persons in the general population will host at least one variant with a MAF < 0.0001. Notably, this is substantially higher than reported rates of ARVC in the general population which makes these variants unlikely to be disease causative (Finocchiaro et al., 2016; Gemayel, Pelliccia, & Thompson, 2001). While studies evaluating this healthy “background” have been essential in understanding the “noise” of the ARVC clinical test, these studies have not provided clarification of secondary genetic findings in WES testing among individuals who were referred for genetic testing due to a myriad of pathologic phenotypes. This is supported by the high burden of incidental variants among ARVC genes within the WES cohort (14% of probands).

**Figure 7:**
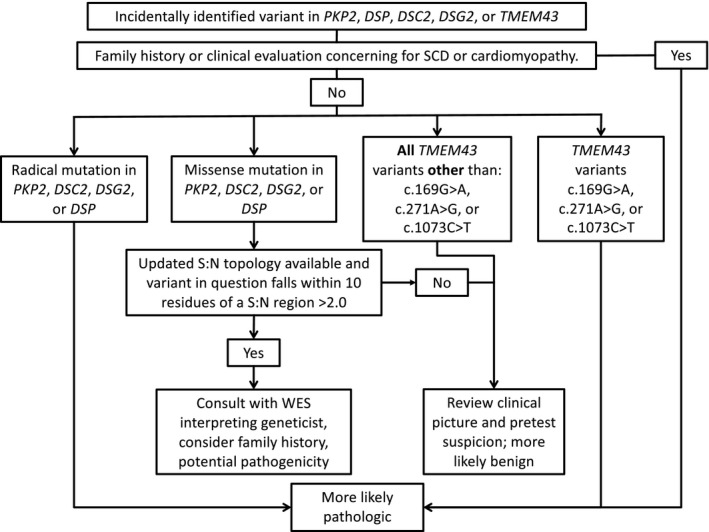
Algorithm for interpretation and reporting of incidentally identified variants in *PKP2*, *DSP*, *DSC2*, *DSG2*, and *TMEM43*. SCD, sudden cardiac death. S:N, signal‐to‐noise. WES, whole exome sequencing

The indication for WES testing and the interpretation of incidental variants is evolving. Current American College of Medical Genetics and Genomics (ACMG) guidelines for the interpretation and reporting of WES results mandate the reporting of incidentally identified variants in genes associated with heritable channelopathies and cardiomyopathies, perpetuating a significant burden of false positives (Green et al., 2017). Given the remarkable similarity between WES and control variants, it is likely that a WES variant found incidentally reflects rare population variation. As such, despite the ACMG recommendations, an incidental WES variant alone cannot serve as a clear diagnostic marker without clinical suspicion for ARVC. Thus, the potentially automatic reporting of incidental variants found in ARVC genes should be refined. At the point of variant interpretation, and prior to assigning pathogenicity, the variant in question must be taken into clinical context given pretest suspicion for ARVC (i.e. personal history, family history, physical exam evidence of ARVC). Further, gene and amino‐acid level analysis of the variant in question, normalized against rare genetic variant frequency at the loci in question, offers one mechanism to differentially weigh the diagnostic relevance of an incidental variant. For example, given the high level of gene‐level S:N for *PKP2* and *DSG2*, variants localizing to these loci may have higher diagnostic relevance. Further, variants falling within the highest peaks of pathogenicity determined by amino acid level S:N analysis may carry more diagnostic weight. Based on these observations, we propose an algorithm to assist in the interpretation of incidental variants in ARVC‐associated genes (Figure 7).

Amino acid‐level S:N analysis among ARVC variants can both distinguish pathologic variants from WES as well as identify functionally relevant protein domains based on localization of pathologic mutations. Within PKP2, the highest areas of pathogenicity occurred in the N‐terminal head region responsible for intracellular binding and interaction with DSG2. It is notable that the majority of contributing variants to this region were radical mutations that resulted in prematurely truncated proteins. In contrast, missense mutations in *PKP2* accounted for significant pathogenic signals correlating to the ARM repeat domains. Plakophilins (PKP1, PKP2, and PKP3) fall under the “*armadillo* plaque protein family,” and commonly have between eight and nine of these rigid, solenoid protein domains, recognized for both their structural roles in interacting with cytoskeleton‐associated proteins and their signaling functions, generating and transducing signals affecting gene expression (Bonné et al., 2003; Hatzfeld, Haffner, Schulze, & Vinzens, 2000; Papagerakis, Shabana, Depondt, Gehanno, & Forest, 2003). Similarly, the S:N analysis of DSG2 demonstrated areas of pathogenicity localizing to protein extracellular regions, specifically the CRDs. Common to all desmogleins is an amino‐terminal extracellular domain that consists of four of these CRDs and the membrane proximal extracellular anchor sequence (Kolegraff, Nava, Laur, Parkos, & Nusrat, 2011). CRDs demonstrate calcium‐dependent adhesion, though the precise mechanism is not well understood (Kline & Mohler, 2013; Sheikh, Ross, & Chen, 2009). Given that this region is likely vital in extracellular desmosomal binding, localization of pathologic mutations to this region supports true variant pathogenicity.

## 5 LIMITATIONS

Although this study represents the largest systematic review of pediatric incidentally‐identified variants among ARVC‐associated genes to date, there are limitations. Our clinical review was retrospective without prospective monitoring of children who host WES variants in ARVC‐associated genes. Further, we recognize that ARVC is infrequently diagnosed early in life, with an average age of symptom onset of ~29‐years‐old, with cardiomyopathic changes most often occurring in adulthood (McNally, MacLeod, & Dellefave‐Castillo^2005^). Thus ARVC may remain clinically occult throughout childhood, and we cannot exclude the possibility that some of the retrospectively reviewed children may yet develop ARVC.

## 6 CONCLUSION

In heritable cardiomyopathy, incidentally identified VUSs in ARVC‐associated genes demonstrate significant similarities with healthy background genetic variation. Incidentally discovered variants in children without a clinical suspicion for ARVC, or cardiomyopathy, are unlikely to represent markers of monogenic disease. Further exploration of genetic “signal‐to‐noise” in large population studies refines the regions of common pathologic involvement for affected proteins and allows for interpretation of ultra‐rare variants.
