# Supplementary figures and images for: Incidentally identified genetic variants in arrhythmogenic right ventricular cardiomyopathy‐associated genes among children undergoing exome sequencing reflect healthy population variation
Source: Mol Genet Genomic Med. 2019 Apr 15;7(6):e593. doi: 10.1002/mgg3.593 (PMC6565596; doi:10.1002/mgg3.593)

**A**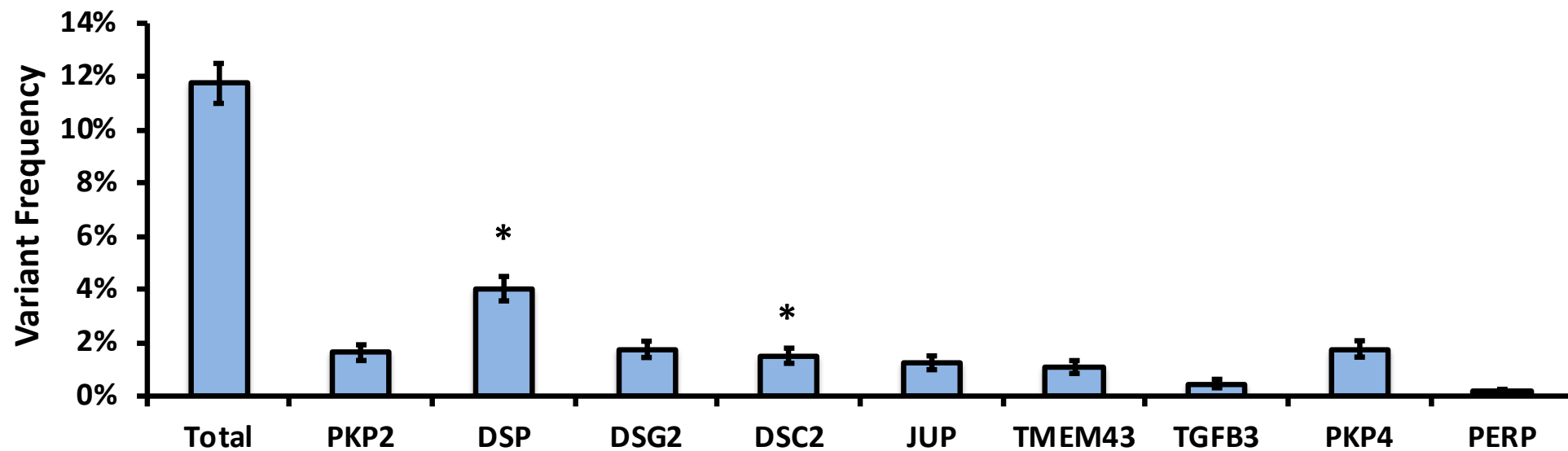**B**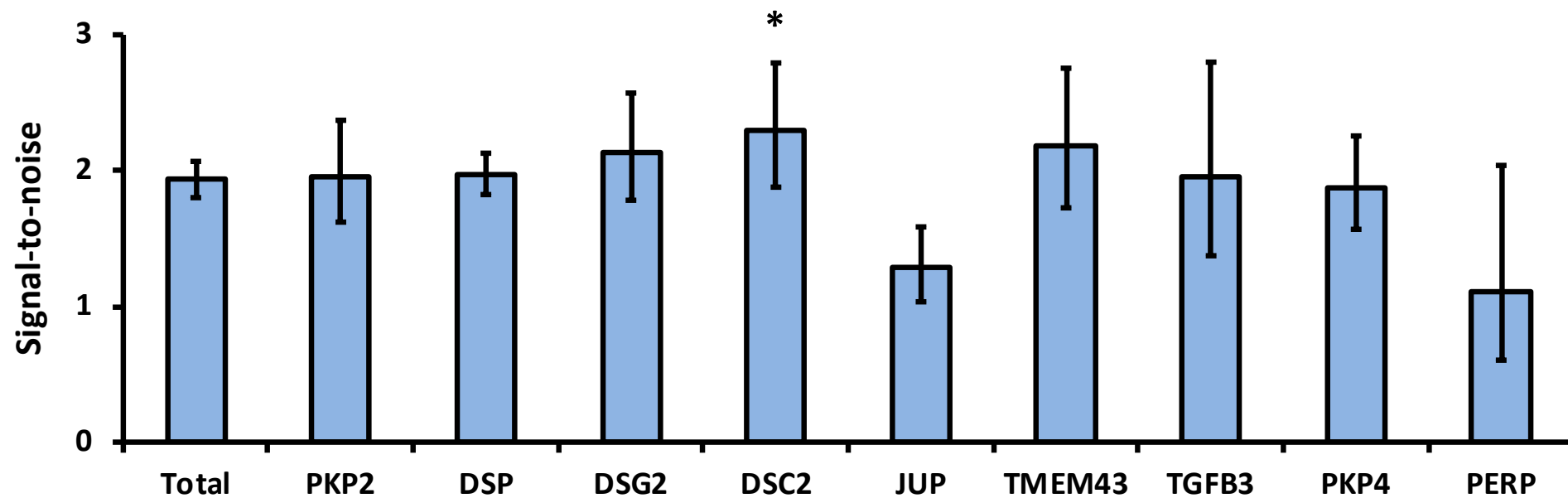**ClinVar-  
verified WES**

|      |      |      |      |      |      |      |      |      |      |
|------|------|------|------|------|------|------|------|------|------|
| 1.93 | 1.96 | 1.97 | 2.14 | 2.29 | 1.28 | 2.18 | 1.96 | 1.88 | 1.11 |
|------|------|------|------|------|------|------|------|------|------|

Supplement: Supplementary file 1 [file MGG3-7-e593-s001.pdf]

**A**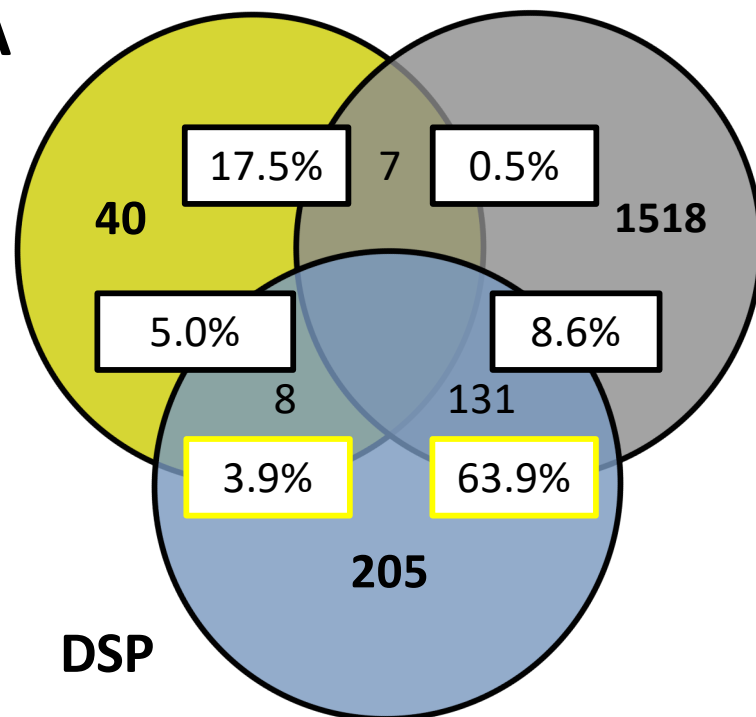

■ ARVC  
■ WES  
■ gnomAD

**B**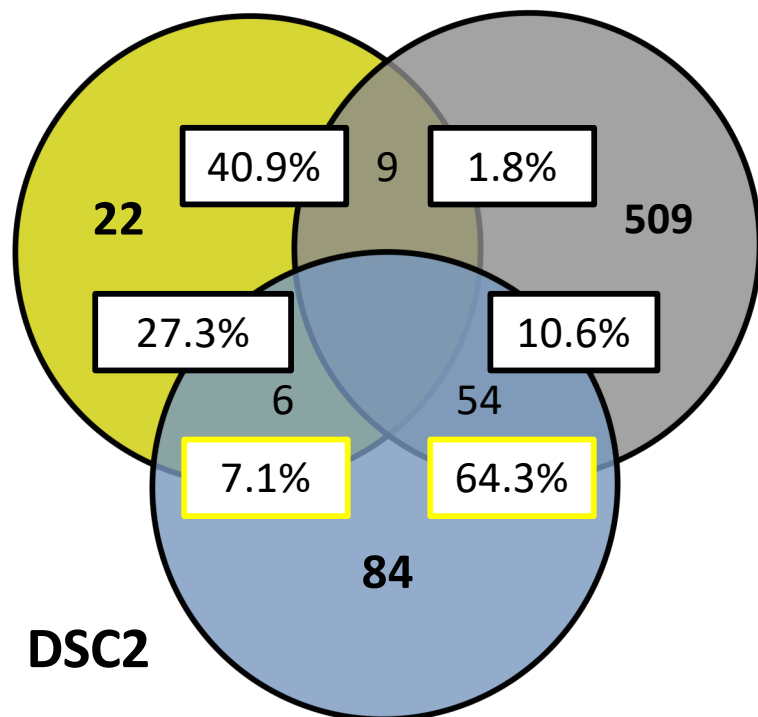**DSC2****C**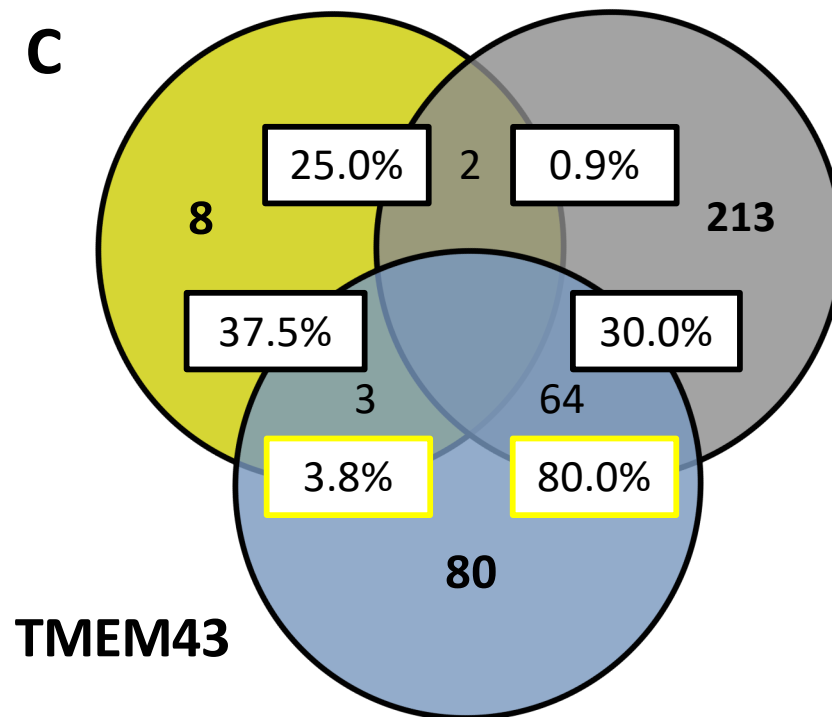**TMEM43**

Supplement: Supplementary file 2 [file MGG3-7-e593-s002.pdf]
