# Supplementary material for: Incidentally identified genetic variants in arrhythmogenic right ventricular cardiomyopathy‐associated genes among children undergoing exome sequencing reflect healthy population variation
Source: Mol Genet Genomic Med. 2019 Apr 15;7(6):e593. doi: 10.1002/mgg3.593 (PMC6565596; doi:10.1002/mgg3.593)
Supplement: Supplementary file 3 [file MGG3-7-e593-s003.docx]

**SUPPLEMENTAL MATERIALS**

**SUPPLEMENTAL METHODS**

**Nomenclature**

For simplicity, the term “variant” is used to denote DNA changes from wildtype which are associated with healthy individuals and incidental findings regardless of whether the result was deemed VUS or “likely pathologic” at the time of WES testing. “Mutation” is used to denote disease-associated DNA changes, such as those identified in individuals with ARVC. “Radical” was used to define variants or mutations that are predicted to cause more than a single amino acid change including nonsense (early termination), insertion/deletion (both in-frame and out-of-frame), and predicted canonical splice site mutations.

**Study Cohorts**

*Whole Exome Sequencing Cohort*

This research study was approved by the Baylor College of Medicine Institutional Review Board. From October 2011 through February 2016, clinical WES was completed at Baylor Genetics Laboratories (Houston, Texas, United States). Sequencing and data analyses were conducted as previously described.(Landstrom et al., 2018) Extracted DNA, subjected to an in-house exome capture platform, VCRome version 2.1 (targeting ~20,000 genes, including the coding and untranslated region (UTR) exons), was sequenced using a HiSeq. This platform has a minimum depth of coverage of 20x with a practical detection rate of ~95% of all single nucleotide variants and insertion/deletions. Samples were additionally analyzed by an Illumina HumanExome-12 v1 cSNP array for quality-control assessment of exome data, as well as for detecting large copy-number variants and regions of absence of heterozygosity.

To establish a background rate of incidentally identified rare variants among clinical next-generation genetic testing cases, rare variants localizing to desmosomal and non-desmosomal ARVC-associated genes were identified in a large cohort of clinical WES referrals. The WES cohort was comprised of individuals referred for genetic testing to the Baylor Genetics Laboratories (Houston, Texas, United States) independent of referral diagnosis or indication for genetic testing. Individuals included in this cohort were genetic testing probands. Individuals excluded from this cohort were 1) non-proband family members, and 2) those whose samples were derived for platform validation studies or from oncological samples. Variants included in this cohort were 1) identified in the coding nucleotide sequence or predicted splice junction of an ARVC-associated gene locus, 2) deemed “likely pathologic” or “variant of undetermined significance” (VUS) at the time of genetic testing according to American College of Medical Genetics and Genomics (ACMG) interpretation guidelines, and 3) included on the clinical report sent to the referring provider as an actionable incidental finding or as a VUS without implications for pathogenicity on an “expanded report” provided to the clinician on request. Variants excluded from this study were 1) interpreted as “not pathogenic” at the time of genetic testing, 2) non-splice site intronic variants, 3) 5’or 3’ untranslated region variants, or 4) synonymous variants.

To adjust for evolution of variant pathogenicity interpretation over time, a ClinVar-verified WES cohort, sub-analysis was performed. This “ClinVar-verified cohort” included all WES cohort variants but excluded those currently designated as “benign” by ClinVar.(Lek et al., 2016)

*ARVC Case Cohort*

An ARVC pathologic case cohort was created for comparison of incidentally identified variants with ostensibly pathologic mutations from the literature. Studies 1) were cohort-based, 2) included cases meeting diagnostic criteria for clinical ARVC, and 3) conducted comprehensive sequencing analysis for *PKP2*, *DSC2*, and *DSG2* at a minimum. To account for evolving interpretations of variant pathogenicity, each variant was referenced against two databases for ARVC-variants, ClinVar and the Cardiogenetics Research Group Database [University Medical Center Gronigen (UCMG)], in a two-tier approach.(Lek et al., 2016; van der Zwaag et al., 2009) Variants were then reclassified as “likely pathologic,” “variant of unknown significance,” or “benign” based on consensus assignment. As the ClinVar database is more recently updated, these classifications were given priority. If not present in ClinVar, the UCMG interpretation was used. Variants absent in both databases received an assignment of VUS. Benign variants were excluded.

*Control Variant Cohort*

A population-based control cohort was established from the publicly available Genome Aggregation Database (gnomAD).(Lek et al., 2016) GnomAD is comprised partly of various disease-specific cohorts in addition to population genetics studies. However, this cohort excludes individuals known to have severe pediatric disease as well as severe disease in their first-degree relatives. Therefore, we utilized these individuals as “control” alleles. Given the importance of establishing an appropriate minor allele frequency (MAF) threshold for rare variant inclusion, we evaluated ClinVar confirmed pathologic ARVC mutations that were also identified in the gnomAD database. We identified the lowest threshold which would exceed the MAF of all pathologic mutations. We set a MAF threshold of <0.0001 for gnomAD rare variant inclusion whereby variants with a greater MAF were excluded from the control cohort. This approach is adapted from previous studies.(Walsh et al., 2017) For the purposes of combined cohort frequencies, *PKP4* and *PERP* were not included given their relative lack of representation across the literature-derived compendium.

*TCH Clinical Cohort*

The Texas Children’s Hospital (TCH) clinical cohort consisted of patients identified by cross-referencing WES cohort referrals and hospital records for individuals who were genotype positive for an ARVC-associated variant and evaluated at TCH. Anonymous information, including basic demographics, personal and family history, transthoracic echocardiograms, electrocardiograms, 24-Holter monitors, genetic testing, and assigned diagnoses were reviewed retrospectively for evidence of cardiomyopathy following exclusion of 1) individuals with structural anomalies of the heart that were hemodynamically significant, 2) individuals with a metabolic or mitochondrial disease that is associated with secondary cardiomyopathic changes, and 3) echocardiograms from patients following cardiac transplantation were excluded, while preceding imaging was included.

**Genetic Analysis and Topologic Mapping**

Desmosomal and non-desmosomal genes associated with ARVC were analyzed. Topology and variant mapping was conducted utilizing consensus primary sequences from Ensembl browser, as indicated, following each respective gene in parentheses.(Hubbard et al., 2005) Unless specifically noted, this included the following genes and encoded proteins: *PKP2*-encoded plakophilin-2 (NM_001005242.2, NP_001005242.2; Gerull et al., 2004), *DSP*-encoded desmoplakin (NM_004415.4, NP_004406.2; Rampazzo et al., 2002), *DSG2*-encoded desmoglein-2 (NM_001943.5, NP_001934.2; Pilichou et al., 2006), *DSC2*-encoded desmocollin-2 (NM_024422.4, NP_077740.1; Syrris et al., 2006), *JUP*-encoded junctional plakoglobin (NM_002230.4, NP_002221.1; McKoy et al., 2000), *TMEM43*-encoded transmembrane protein-43 (NM_024334.2, NP_077310.1; Merner et al., 2008), *TGFB3*-encoded transforming growth factor beta-3 (NM_003239.4, NP_001316868.1; Beffagna et al., 2005), *PKP4*-encoded plakophilin-4 (NM_003628.4, NP_003619.2; Gandjbakhch et al., 2013), *PERP*-encoded p53 effector related to PMP22 (NM_022121.5, NP_071404.2; Gandjbakhch et al., 2013). For comparisons of global cohort frequency, *PKP4* and *PERP* were not included given the absence of robust evidence in multiple studies for association with ARVC.(Gandjbakhch et al., 2013)

**Gene- and Amino Acid-Level Signal-to-Noise Calculation**

For gene-level signal-to-noise (S:N) calculations, variant frequency for each gene for the WES and ARVC cohorts was normalized against the corresponding gene frequency in the control cohort. For amino acid-level signal-to-noise calculations, frequency of a WES or ARVC cohort-associated variant at a given amino acid position was calculated. A rolling average of these frequencies was normalized against a similarly calculated frequency for a corresponding amino acid from the control cohort. The resultant value was mapped along the primary sequence with linear topology overlaid.

**Statistics**

Statistical results were expressed as mean with variance expressed as standard deviation or median and interquartile range (brackets), as appropriate. Variance of prevalence/proportion was expressed as the exact 95% confidence interval around proportion when statistical comparisons were made (brackets). Comparisons were made by Chi-Square with Yates Correction using OpenEpi.(Dean et al., 2006) The statistical significance threshold was set at *P*<0.05.

**SUPPLEMENTAL RESULTS**

**Clinical WES Variants Hosted by Multiple Individuals**

Given the presence of founder mutations among ARVC cases, we explored whether variants were seen in multiple individuals with the WES cohort. There were 707 unique variants identified in the WES cohort, of which 679 (96.0% [94.6-97.5] of total unique variants) were VUS, and 28 (4.0% [2.5-5.4]) were pathologic. Overall, 152 (21.5% [18.5-24.5]) of all WES variants were identified in more than one WES proband. Of the unique variants, 5 (0.7% [0.1-1.3]), occurred ≥10 times. These variants are detailed in **Supplemental Table 2**. Of note, none of these variants were reported as pathogenic at the time of genetic test reporting. Further, none of these commonly occurring variants from the WES cohort were present in the ARVC case cohort.

**ARVC Case Cohort**

The ARVC case cohort was comprised of 14 studies derived from the literature which met inclusion and exclusion criteria.(Asimaki et al., 2009; Bao et al., 2013; Baskin et al., 2013; Bhuiyan et al., 2009; Cox et al., 2011; den Haan et al., 2009; Fressart et al., 2010; Gandjbakhch et al., 2013; Kapplinger et al., 2011; Klauke et al., 2010; La Gerche et al., 2010; Ohno et al., 2013; Tan et al., 2010; Xu et al., 2010)

**ClinVar-Verified WES Cohort Gene Frequencies and Signal-to-Noise**

Following re-classification of WES cohort variants by ClinVar designations, and exclusion of consensus “benign” variants, the cohort combined variant frequency had a modest reduction to 11.7% [11.0-12.5] and variants localized most commonly to *DSP* (4.0% [3.6-4.5]), *PKP4* (1.8% [1.5-2.1]), and *DSG2* (1.8% [1.4-2.1]). These results are summarized in **Supplemental Figure 1**. Several of these re-classifications resulted in variant frequency reductions that were statistically significant, to include the combined cohort variant frequency, the variant frequency of *DSC2*, and that of *DSP*; the latter two were the result of the re-classification of 66 *DSC2* and 51 *DSP* variants as benign. No other gene-specific variant frequencies exhibited significant reduction.

Given the reductions in gene frequency in the ClinVar-verified WES cohort, we next explored how these changes affected S:N. Reductions were seen in respective S:N ratios across the ClinVar-verified WES cohort as compared to the original WES cohort. The combined S:N reduced to 1.93 [1.80-2.07]. The genes displaying the greatest S:N were *DSC2*, *TMEM43*, and *DSG2* at 2.29 [1.88-2.79], 2.18 [1.73-2.75], and 2.14 [1.78-2.57], respectively. The only gene-specific S:N ratio to show significant reduction was *DSC2*. All genes showed S:N ratios that were significantly equivalent to, or less than, 2.0. Only S:N ratios for the genes *JUP* and *PERP* were significantly indistinguishable from 1.0.

**Affected Amino Acid Position for DSP, DSC2, and TMEM43**

For DSP, the WES cohort variants localized to 205 unique amino acid positions. ARVC case variants occupied 40 positions while the control variants occupied 1518. 63.9% [57.3-70.5] of the WES cohort positions were shared with the control cohort, while only 3.9% [1.3-6.6] of the WES cohort positions were shared with ARVC cases. For DSC2, the WES cohort demonstrated 84 uniquely affected positions, the ARVC cohort 22, and the control cohort 509. 64.3% [54.0-74.5] of affected positions in the WES were common to those in the control, compared to only 7.1% [1.6-12.6] that were shared with those in pathologic cases. Lastly, for TMEM43, the WES cohort had 80 unique affected positions, the ARVC 8, and the control 213. 80.0% [71.2-88.8] of the WES variants were common with those present in the control cohort, compared to only 3.8% [0.0-7.9] that were shared with the case cohort.

**Mutations contributing to PKP2 regions of greatest signal in ARVC cohort**

The first large pathogenic signal occurred between amino acid positions 74 and 92, with a peak at 84. The biggest contributor to this was a frequent occurrence of p.Arg79X, the result of a c.235C>T mutation resulting in a stop codon and yielding a prematurely truncated protein. A similar large signal was seen ranging from amino acid positions 128 to 145, with a peak at 138, resulting largely from a frequent occurrence of another prematurely truncated protein, p.Gln133X, resulting from a c.397C>T nonsense mutation. These two mutations have been previously described, as recurrent and representing a founder effect in the Dutch population.(van der Zwaag et al., 2010; van Tintelen et al., 2006) In the control cohort, the former occurred at a MAF of 4.06E-6, with only 1 reported occurrence, in the European (Non-Finnish) subset. The latter was not found. Neither of these mutations was present in the WES cohort.

**Mutations contributing to WES cohort DSG2 region of signal**

One area of concentrated signal was seen on the DSG2 topology of the WES cohort, beginning at amino acid position 287 and continuing to 299, with a peak at 296. This was due largely to 5 missense variants in the region. When referenced against ClinVar, three maintained their classification as VUS, and the remaining two did not have reported classifications. Only one, a c.889G>A VUS missense mutation yielding a D297N protein change, was found in the ARVC cohort. These mutations are detailed in **Supplemental Table 3**.

**WES cohort subjects with 3 or more variants**

Of 3 subjects from the WES cohort with 3 or more variants, 2 were seen and evaluated at TCH. The first was a 4-year-old female with a Rag1 variant induced severe combined immunodeficiency disease (SCID), now status post bone marrow transplant. Echos have been performed given her history of SCID and chemotherapy, and no cardiac abnormalities have been noted to date. The second patient is a 13-year-old male with *TTN*-variant induced titinopathy, who has been regularly followed by cardiology and shown progressive decline in left ventricular systolic function. These patients’ relevant clinical data are detailed in **Supplemental Table 4**.

**SUPPLEMENTAL REFERENCES**

Asimaki, A., Tandri, H., Huang, H., Halushka, M. K., Gautam, S., Basso, C., Saffitz, J. E. (2009). A new diagnostic test for arrhythmogenic right ventricular cardiomyopathy. *N Engl J Med, 360*(11), 1075-1084. doi:10.1056/nejmoa0808138

Bao, J., Wang, J., Yao, Y., Wang, Y., Fan, X., Sun, K., Song, L. (2013). Correlation of ventricular arrhythmias with genotype in arrhythmogenic right ventricular cardiomyopathy. *Circ Cardiovasc Genet, 6*(6), 552-556. doi:10.1161/circgenetics.113.000122

Baskin, B., Skinner, J. R., Sanatani, S., Terespolsky, D., Krahn, A. D., Ray, P. N., Hamilton, R. M. (2013). TMEM43 mutations associated with arrhythmogenic right ventricular cardiomyopathy in non-Newfoundland populations. *Hum Genet, 132*(11), 1245-1252. doi:10.1007/s00439-013-1323-2

Beffagna, G., Occhi, G., Nava, A., Vitiello, L., Ditadi, A., Basso, C., Rampazzo, A. (2005). Regulatory mutations in transforming growth factor-beta3 gene cause arrhythmogenic right ventricular cardiomyopathy type 1. *Cardiovasc Res, 65*(2), 366-373. doi:10.1016/j.cardiores.2004.10.005

Bhuiyan, Z. A., Jongbloed, J. D., van der Smagt, J., Lombardi, P. M., Wiesfeld, A. C., Nelen, M., van Tintelen, J. P. (2009). Desmoglein-2 and desmocollin-2 mutations in dutch arrhythmogenic right ventricular dysplasia/cardiomypathy patients: results from a multicenter study. *Circ Cardiovasc Genet, 2*(5), 418-427. doi:10.1161/circgenetics.108.839829

Cox, M. G., van der Zwaag, P. A., van der Werf, C., van der Smagt, J. J., Noorman, M., Bhuiyan, Z. A., Hauer, R. N. (2011). Arrhythmogenic right ventricular dysplasia/cardiomyopathy: pathogenic desmosome mutations in index-patients predict outcome of family screening: Dutch arrhythmogenic right ventricular dysplasia/cardiomyopathy genotype-phenotype follow-up study. *Circulation, 123*(23), 2690-2700. doi:10.1161/circulationaha.110.988287

Dean, A., Sullivan, K., & Soe, M. (2006). OpenEpi: Open Source Epidemiologic Statistics for Public Health, version 3.01. Retrieved from [www.OpenEpi.com](file:///Users/andrew_landstrom/Library/Containers/com.apple.mail/Data/Library/Mail%20Downloads/77E8145B-FB02-494F-AAFD-39EDDC141B27/www.OpenEpi.com)

den Haan, A. D., Tan, B. Y., Zikusoka, M. N., Ll adó, L. I., Jain, R., Daly, A., Judge, D. P. (2009). Comprehensive desmosome mutation analysis in north americans with arrhythmogenic right ventricular dysplasia/cardiomyopathy. *Circ Cardiovasc Genet, 2*(5), 428-435. doi:10.1161/circgenetics.109.858217

Fressart, V., Duthoit, G., Donal, E., Probst, V., Deharo, J. C., Chevalier, P., Charron, P. (2010). Desmosomal gene analysis in arrhythmogenic right ventricular dysplasia/cardiomyopathy: spectrum of mutations and clinical impact in practice. *Europace, 12*(6), 861-868. doi:10.1093/europace/euq104

Gandjbakhch, E., Vite, A., Gary, F., Fressart, V., Donal, E., Simon, F., Villard, E. (2013). Screening of genes encoding junctional candidates in arrhythmogenic right ventricular cardiomyopathy/dysplasia. *Europace, 15*(10), 1522-1525. doi:10.1093/europace/eut224

Gerull, B., Heuser, A., Wichter, T., Paul, M., Basson, C. T., McDermott, D. A., Thierfelder, L. (2004). Mutations in the desmosomal protein plakophilin-2 are common in arrhythmogenic right ventricular cardiomyopathy. *Nat Genet, 36*(11), 1162-1164. doi:10.1038/ng1461

Hubbard, T., Andrews, D., Caccamo, M., Cameron, G., Chen, Y., Clamp, M., Birney, E. (2005). Ensembl 2005. *Nucleic Acids Res, 33*(Database issue), D447-453. doi:10.1093/nar/gki138

Kapplinger, J. D., Landstrom, A. P., Salisbury, B. A., Callis, T. E., Pollevick, G. D., Tester, D. J., Ackerman, M. J. (2011). Distinguishing arrhythmogenic right ventricular cardiomyopathy/dysplasia-associated mutations from background genetic noise. *J Am Coll Cardiol, 57*(23), 2317-2327. doi:10.1016/j.jacc.2010.12.036

Klauke, B., Kossmann, S., Gaertner, A., Brand, K., Stork, I., Brodehl, A., Milting, H. (2010). De novo desmin-mutation N116S is associated with arrhythmogenic right ventricular cardiomyopathy. *Hum Mol Genet, 19*(23), 4595-4607. doi:10.1093/hmg/ddq387

La Gerche, A., Robberecht, C., Kuiperi, C., Nuyens, D., Willems, R., de Ravel, T., Heidbüchel, H. (2010). Lower than expected desmosomal gene mutation prevalence in endurance athletes with complex ventricular arrhythmias of right ventricular origin. *Heart, 96*(16), 1268-1274. doi:10.1136/hrt.2009.189621

Landstrom, A. P., Fernandez, E., Rosenfeld, J. A., Yang, Y., Dailey-Schwartz, A. L., Miyake, C. Y., Kim, J. J. (2018). Amino acid-level signal-to-noise analysis of incidentally identified variants in genes associated with long QT syndrome during pediatric whole exome sequencing reflects background genetic noise. *Heart Rhythm*. doi:10.1016/j.hrthm.2018.02.031

Lek, M., Karczewski, K. J., Minikel, E. V., Samocha, K. E., Banks, E., Fennell, T., Consortium, E. A. (2016). Analysis of protein-coding genetic variation in 60,706 humans. *Nature, 536*(7616), 285-291. doi:10.1038/nature19057

McKoy, G., Protonotarios, N., Crosby, A., Tsatsopoulou, A., Anastasakis, A., Coonar, A., McKenna, W. J. (2000). Identification of a deletion in plakoglobin in arrhythmogenic right ventricular cardiomyopathy with palmoplantar keratoderma and woolly hair (Naxos disease). *Lancet, 355*(9221), 2119-2124. doi:10.1016/S0140-6736(00)02379-5

Merner, N. D., Hodgkinson, K. A., Haywood, A. F., Connors, S., French, V. M., Drenckhahn, J. D., Young, T. L. (2008). Arrhythmogenic right ventricular cardiomyopathy type 5 is a fully penetrant, lethal arrhythmic disorder caused by a missense mutation in the TMEM43 gene. *Am J Hum Genet, 82*(4), 809-821. doi:10.1016/j.ajhg.2008.01.010

Ohno, S., Nagaoka, I., Fukuyama, M., Kimura, H., Itoh, H., Makiyama, T., Horie, M. (2013). Age-dependent clinical and genetic characteristics in Japanese patients with arrhythmogenic right ventricular cardiomyopathy/dysplasia. *Circ J, 77*(6), 1534-1542. doi: 10.1016/j.joa.2016.01.006

Pilichou, K., Nava, A., Basso, C., Beffagna, G., Bauce, B., Lorenzon, A., Rampazzo, A. (2006). Mutations in desmoglein-2 gene are associated with arrhythmogenic right ventricular cardiomyopathy. *Circulation, 113*(9), 1171-1179. doi:10.1161/circulationaha.105.583674

Rampazzo, A., Nava, A., Malacrida, S., Beffagna, G., Bauce, B., Rossi, V., Danieli, G. A. (2002). Mutation in human desmoplakin domain binding to plakoglobin causes a dominant form of arrhythmogenic right ventricular cardiomyopathy. *Am J Hum Genet, 71*(5), 1200-1206. doi:10.1086/344208

Syrris, P., Ward, D., Evans, A., Asimaki, A., Gandjbakhch, E., Sen-Chowdhry, S., & McKenna, W. J. (2006). Arrhythmogenic right ventricular dysplasia/cardiomyopathy associated with mutations in the desmosomal gene desmocollin-2. *Am J Hum Genet, 79*(5), 978-984. doi:10.1086/509122

Tan, B. Y., Jain, R., den Haan, A. D., Chen, Y., Dalal, D., Tandri, H., Judge, D. P. (2010). Shared desmosome gene findings in early and late onset arrhythmogenic right ventricular dysplasia/cardiomyopathy. *J Cardiovasc Transl Res, 3*(6), 663-673. doi:10.1007/s12265-010-9224-4

van der Zwaag, P. A., Cox, M. G., van der Werf, C., Wiesfeld, A. C., Jongbloed, J. D., Dooijes, D., van Tintelen, J. P. (2010). Recurrent and founder mutations in the Netherlands : Plakophilin-2 p.Arg79X mutation causing arrhythmogenic right ventricular cardiomyopathy/dysplasia. *Neth Heart J, 18*(12), 583-591. doi: 10.1007/s12471-010-0839-5

van der Zwaag, P. A., Jongbloed, J. D., van den Berg, M. P., van der Smagt, J. J., Jongbloed, R., Bikker, H., van Tintelen, J. P. (2009). A genetic variants database for arrhythmogenic right ventricular dysplasia/cardiomyopathy. *Hum Mutat, 30*(9), 1278-1283. doi:10.1002/humu.21064

van Tintelen, J. P., Entius, M. M., Bhuiyan, Z. A., Jongbloed, R., Wiesfeld, A. C., Wilde, A. A., Hauer, R. N. (2006). Plakophilin-2 mutations are the major determinant of familial arrhythmogenic right ventricular dysplasia/cardiomyopathy. *Circulation, 113*(13), 1650-1658. doi:10.1161/circulationaha.105.609719

Walsh, R., Thomson, K. L., Ware, J. S., Funke, B. H., Woodley, J., McGuire, K. J., Watkins, H. (2017). Reassessment of Mendelian gene pathogenicity using 7,855 cardiomyopathy cases and 60,706 reference samples. *Genet Med, 19*(2), 192-203. doi:10.1038/gim.2016.90

Xu, T., Yang, Z., Vatta, M., Rampazzo, A., Beffagna, G., Pilichou, K., Investigators, M. S. o. R. V. D. (2010). Compound and digenic heterozygosity contributes to arrhythmogenic right ventricular cardiomyopathy. *J Am Coll Cardiol, 55*(6), 587-597. doi:10.1016/j.jacc.2009.11.020

**SUPPLEMENTAL TABLES**

**Supplemental Table 1: WES cohort demographics**

|  | Total Cohort | Variant-Positive Individuals |
| --- | --- | --- |
| Total Individuals | 7244 | 1018 |
| Total Families | 7066 | 1018 |
| Total Probands | 7066 | 1018 |
| Male | 3909 (54.0%) | 540 (53.0%) |
| Female | 3274 (45.2%) | 468 (46.0%) |
| Fetal | 61 (0.8%) | 10 (1.0%) |
| Age at genetic testing | 9.4 [1d -91y] | 9.6 [1d-84y] |
| Unique Variants | 707 | |
| VUS | 679 (96.0% [94.6-97.5]) | |
| Pathologic | 28 (4.0% [2.5-5.4]) | |
| Variant positive probands | 1018 | |
| Single | 938 | |
| Double | 77 | |
| Triple | 3 | |

VUS, variant of unknown significance.

**Supplemental Table 2: Most frequently occurring variants in WES Cohort**

| Gene | DNA Change | Protein Change | WES  Designation | Occur | Frequency in  WES Cohort | Present in ARVC Cases | ClinVar Classification |
| --- | --- | --- | --- | --- | --- | --- | --- |
| *TMEM43* | c.547G>A | p.G183S | VUS | 10 | 0.14%  [0.05-0.23] | No | *Not reported* |
| *PKP4* | c.572C>T | p.A191V | VUS | 12 | 0.17%  [0.07-0.27] | No | *Not reported* |
| *DSC2* | c.2368_2370del | p.790* | VUS | 17 | 0.24%  [0.13-0.35] | No | VUS |
| *DSP* | c.8110_8112del | p.2704* | VUS | 23 | 0.33%  [0.19-0.46] | No | *Not reported* |
| *DSC2* | c.2688_2689insGA | p.E896fs | Likely path/VUS† | 42 | 0.59%  [0.42-0.77] | No | Benign |

ARVC, arrhythmogenic right ventricular cardiomyopathy; del, deletion; fs, frameshift; ins, insertion; occur, number of occurrences in the cohort; VUS, variant of unknown significance; WES, whole exome sequencing; †, variant pathogenicity designation was interpreted as both likely pathologic and VUS.

**Supplemental Table 3: WES cohort *DSG2* variants contributing to areas of increased signal:noise**

| DNA change | Protein Change | # of WES subjects | ClinVar Interpretation | MAF in  gnomAD |
| --- | --- | --- | --- | --- |
| c.880A>G | K294E | 3 | VUS | 2.17E-05 |
| c.875G>A | R292H | 4 | VUS | 8.30E-05 |
| c.889G>A | D297N | 1 | VUS | 1.08E-05 |
| c.862G>A | V288I | 2 | *Not reported* | 4.69E-05 |
| c.902T>C | I301T | 1 | *Not reported* | *Not present* |

VUS, variant of unknown significance; WES, whole exome sequencing.

**Supplemental Table 4: TCH Cohort subject with at least 3 positive variants**

| Age (y), Sex,  Race | Gene involved | Variant | Protein consequence | Variant type & variant class | #  Echo  EKG  Holter | Follow-up (y) | Organ system prompting WES |
| --- | --- | --- | --- | --- | --- | --- | --- |
| 4.5, F, C | *DSP*  *JUP*  *DSC2* | c.478C>T  c.152G>T  c.302C>A | p.R160X  p.R51L  p.T101N | VUS; radical  VUS; missense  VUS; missense | 4  7  0 | 3.31 | Immunology |
| 13.4, M, C | *TMEM43*  *DSP*  *DSG2* | c.918G>T  c.5935C>G  c.3244C>G | p.M306I  p.Q1979E  p.P1082A | VUS; missense  VUS; missense  VUS; missense | 4  3  2 | 5.77 | Neurology |

C, Caucasian non-Hispanic; F, female; M, male; VUS, variant of unknown significance; WES, whole exome sequencing.

**Supplemental Table 5: Clinical characteristics of TCH cohort subjects with cardiomyopathy**

| **Demo** Gender Age Race | **Echo and MRI** | **Cardiac Diagnoses** | **Variant & variant category** | **Gene** | **DNA Change** | **Amino Acid Change** | **# of**  **Echos**  **ECGs**  **Holters** | **Echo follow up (years)** | **H/o VT or**  **SCD**  **(Y/N)** | **Involved organ systems prompting WES** | **Deceased (Y/N)** | **Pertinent variants on WES** |
| --- | --- | --- | --- | --- | --- | --- | --- | --- | --- | --- | --- | --- |
| Male 3.85 years Caucasian | **Echo:**  - HCM - LAD **MRI:** - Subvalvular LV outflow tract obstruction - no fibrofatty replacement | - HOCM | VUS, Missense VUS, Missense | DSP DSP | c.157T>G c.12C>G | p.S53A p.N4K | 28  19  1 | 8.82 | N  N | Genetics (metabolic) Cardiology | N | ***TTN*** VUS: 3002T>C, 8525T>C, 32191G>A |
| Male 5.40 years Caucasian | **Echo:** - Globular LVD with systolic failure  - RVD | - Acute RV heart failure  - LVNC  - Chronic systolic heart failure  - LV systolic failure | VUS, Missense | PKP2 | c.1636G>A | p.A546T | 41  43  13 | 7.50 | Y  N | Cardiology Connective tissue (vascular) | N | ***SCN5A*** Pathogenic: 5227G>A |
| Female 4.88 years African American | **Echo:**  - LVD - LAD - RVD | - CHF  - Biventricular dysfunction  - DCM | VUS, Missense | PKP2 | c.914G>A | p.G305E | 4  4  0 | 0.02 | N  N | Cardiology | Y | ***MYBPC3*** VUS: 3190+4C>T  ***TTN*** VUS: 28454C>T, 38993-5A>G, 90056G>A, 18988A>G |
| Male 18.68 years Caucasian | **Echo:**  - HCM - Localized septal hypertrophy | - HCM  - Single chamber pacemaker | VUS, Missense | DSP | c.4490G>A | p.R1497Q | 13  57  32 | 8.01 | N  N | Neurology | N | ***TTN*** VUS: 5231C>T, 79912_79914delGAA |
| Male 15.18 years Caucasian | **Echo:** - LVNC  - LVD - LAD | - LVNC  - RV systolic failure  - CHF | VUS, Missense | DSP | c.8455A>C | p.M2819L | 16  22  2 | 0.45 | Y  N | Cardiology | Y | ***LAMP2*** Pathogenic: 929-1G>A  ***ACTN2*** VUS: 1670C>T |
| Female 6.45 years African American | **Echo:** - RV systolic failure - Biatrial dilation | - Chronic biventricular systolic and diastolic heart failure  - DCM  - Cardiac pacemaker | Likely path, Radical | PKP2 | c.144_165del | p.Q49fs | 48  29  6 | 3.48 | N  Y | Cardiology | Y | ***BAG3*** VUS: 25A>G  ***JPH2*** VUS: 1808C>T  ***TTN*** VUS: 52145G>A  ***DMD*** VUS: 5909A>G |
| Male 1.39 years African American | **Echo:** - LVD - LV systolic failure - LVH | - DCM | Likely path, Missense | PKP2 | c.663C>A | p.Y221X | 49  18  2 | 5.41 | N  Y | Cardiology | N | ***MYPN*** VUS: 12543G>A  ***MYOM1***  VUS: 3412C>T  ***TTN*** VUS: 86452G>A, 35942A>C, 14944C>T, 12834G>T, 31495C>A, 80636C>G, 79433T>G, 12128C>T  ***MYBPC3*** VUS: 3004C>T |
| Male 149 days Caucasian | **Echo:**  - LAD | None | VUS, Missense | DSP | c.8455A>C | p.M2819L | 7  33  14 | 3.11 | N  N | Neurology | N | ***MYPN*** VUS: 1900G>A |

CHF, congestive heart failure; DCM, dilated cardiomyopathy; HCM/HOCM, hypertrophic (obstructive) cardiomyopathy; H/o, history of; LAD, left atrial dilation; LV, left ventricle; LVD, left ventricular dilation; LVH, left ventricular hypertrophy; LVNC, left ventricular non-compaction; RVD, right ventricular dilation; SCD, sudden cardiac death; VUS, variant of unknown significance; VT, ventricular tachycardia; WES, whole exome sequencing.

**SUPPLEMENTAL FIGURE LEGENDS**

**Figure 1. A,** Bar graph of gene-specific frequencies for WES Cohort following exclusion of variants designated as benign by ClinVar. **B,** Bar graph of S:N gene-specific frequency analysis for the ClinVar-verified WES Cohort. *, *P* < 0.05 compared to corresponding value in original WES cohort (see Figures 2B and 3). WES, whole exome sequencing.

**Figure 2. A,** Variant localized amino acid overlap by cohort for DSC2. **B,** Variant localized amino acid overlap by cohort for DSP. **C,** Variant localized amino acid overlap by cohort for TMEM43. ARVC, arrhythmogenic right ventricular cardiomyopathy. WES, whole exome sequencing.
